# New York Society of Dental Surgeons

**Published:** 1854-01

**Authors:** 


### NEW YORK SOCIETY OF DENTAL SURGEONS.

This society held its last annual meeting on Tuesday, the 13th of Sept. last, in New York. We see but a brief report of its proceedings in the September number of the Recorder. The following officers were elected for the present year: President, Martin K. Bridges; 1st Vice President, Benjamin Lord; 2d do. A Johnson; Recording Secretary, Lemuel Covell; Corresponding Secretary, Chas. C. Allen; Treasurer, Benj. J. Maguire; Librarian, E. F. Arnoux.

